# The outcomes of the management of complex distal tibia and ankle fractures in elderly with tibiotalocalcaneal nail in a minimum 12-month follow-up period

**DOI:** 10.1007/s00590-024-03970-2

**Published:** 2024-05-18

**Authors:** Georgios Kotsarinis, Emanuele Santolini, Nikolaos Kanakaris, Peter V. Giannoudis

**Affiliations:** 1 Academic Department of Trauma and Orthopaedic Surgery, School of Medicine, Clarendon Wing, Leeds General Infirmary, University of Leeds, Floor D, Great George Street, Leeds, LS1 3EX UK; 2 Orthopaedics and Trauma Unit, IRCCS Ospedale Policlinico San Martino, Genoa, Italy; 3 NIHR Leeds Biomedical Research Center, Chapel Allerton Hospital, Leeds, UK

**Keywords:** Tibiotalocalcaneal nail, Hindfoot nail, Complex ankle fractures, Complex distal tibia fractures, Pilon fractures

## Abstract

**Purpose:**

To evaluate the clinical outcomes of the use of tibiotalocalcaneal nail for the treatment of complex distal tibia and ankle fractures in elderly people, in a major trauma centre.

**Methods:**

Elderly patients (age > 65) with distal tibia or ankle fractures that underwent stabilization with a tibiotalocalcaneal nail were eligible to participate. Exclusion criteria were patients that died or were lost to follow-up and cases in which the nail was used in a chronic setting, such as malunion and non-union. Main parameters evaluated were fracture union, complications and functional outcomes. The functional outcome was assessed using the Olerud–Molander Ankle Score (OMAS). The minimum follow-up was 12 months.

**Results:**

Thirty-two consecutive patients (12 males) with a mean age of 80.2 years (range 66–98) met the inclusion criteria and formed the basis of this study. Fracture union was achieved in 93.8% of the cases at a mean time of 3.9 months (range 2–8). Two patients developed surgical site infections and underwent reoperation before union. The overall complication rate was 25.1%, while the respective reintervention rate was 18.8%. In terms of functional outcomes, the mean OMAS score was 45, ranging from 20 to 70.

**Conclusion:**

Tibiotalocalcaneal nailing can be considered as an acceptable less invasive option with good functional outcomes for the treatment of complex distal tibia and ankle fractures in frail patients with problematic local soft tissues.

## Introduction

Ankle and distal tibia fractures account for 9% and 0.7% of all fractures in adults, respectively, with their incidence increasing proportionally with age [1]. The main treatment goal of intra-articular fractures is precise anatomical reduction, stable fixation and prevention of posttraumatic arthritis. However, in low demand, poorly ambulated frail patients with complex distal tibia or ankle fractures, the main target is stabilization of the fracture and facilitation of early weight bearing [2, 3], and avoidance of secondary complications given the high incidence of poor bone stock and soft tissue quality in such patients [4]. While the management of these cases includes various treatment options, special consideration of the host’s comorbidities, level of activity and the fracture pattern should guide the surgeon towards an individualized treatment [2, 3, 5].

Retrograde hindfoot nailing (tibiotalocalcaneal nail, TTCN) is considered to be advantageous compared to other treatment options in patients with bone fragility, and poor skin quality, as well as in those who are unable to comply with prescription of limited weight-bearing postoperative protocols [2, 4, 5]. However, in younger individuals limited evidence is available with one study highlighting concerns due to the unnecessary sacrifice of the subtalar joint and the long-term sequel of hindfoot fusion in active patients [6].

The purpose of this study therefore was to evaluate the clinical outcomes of the use of tibiotalocalcaneal nail for the treatment of complex distal tibia and ankle fractures in elderly patients being managed at a Major Trauma Centre in the UK.

## Patients and methods

Following approval from the Institutional Review Board, a retrospective consecutive series study was conducted in our institution, in order to assess the results of the use and the effectiveness of TTCN in the treatment of distal tibia and ankle fractures. Between January 2013 and December 2020, all elderly patients [7] with distal tibia and ankle fractures managed with the Phoenix Ankle Arthrodesis Nail System (Zimmer Biomet, Indiana, USA) were eligible to participate in this study. Cases in which the nail was used in a chronic setting, such as for malunions and non-unions, and cases that died or were lost to follow-up were excluded from the study. Such details were collected and analysed as patients demographics (age, gender), mechanism of injury, AO fracture classification [8], additional injuries, type of fracture (open/closed), time to union, implant-related complications and implant removal reasons.

Emphasis was given to the following two parameters:

1. Time to fracture union assessed both clinically and radiographically. Clinical union was defined as the ability of the patient to full weight bear without pain, whereas radiographic union was defined as cortical apposition at 3 out of 4 cortices at the fracture site [9].
2. Incidence and frequency of implant- or technique-related complications.

Functional outcome evaluation at the time of last follow-up was carried out using the Olerud–Molander Ankle Score (OMAS) [10].

### Protocol and technique of stabilization

Upon presentation at the emergency department, all open fractures were administered of intravenous 1.2 g amoxicillin and clavulanate (or 600 mg clindamycin if allergic to penicillin), according to the local Trust protocol for maximum 72 h or less depending on the definitive management of soft tissue injury. On admission, all patients had their fracture reduced and placed in a back slab (plaster of Paris) for comfort and support. CT scanning was acquired in all cases with distal tibia fractures. Affected extremities were kept elevated for control of the soft tissue swelling and underwent regular observations for neurovascular assessment.

All operations were carried out under general or spinal anaesthesia, with a prophylactic preoperative antibiotic dose (flucloxacillin and gentamycin for closed fractures or teicoplanin and gentamycin for open fractures) administered at induction. After standard skin preparation with chlorhexidine solution and sterile draping below the knee, a tourniquet was usually (60% in this series) inflated with the patient in supine position on a radiolucent table.

The entry point was identified by obtaining routine AP and lateral radiographs of the ankle joint. After a 2 cm longitudinal incision was performed on the plantar aspect of the heel and the entry point was prepared, a guidewire was advanced through the calcaneus, body of the talus and distal tibia under image intensifier fluoroscopic guidance. At the same time, the assistant was stabilizing the hindfoot in the optimal fusion position with the fracture reduced. Once desired position confirmed, reaming over the guidewire was initiated, while reduction was maintained. The appropriate diameter of the nail was determined as 1 mm less than the last reamer head size used. All but one nail did not extend the isthmus of the tibia.

Postoperatively, all patients were prescribed thromboprophylaxis tinzaparin 3500 IU for a period of 6 weeks. All patients were encouraged to full weight bearing in an air cast boot using elbow crutches or a Zimmer Frame, unless contraindicated by concomitant injuries.

Following discharge from the hospital, patients were followed up at the outpatient clinic at regular intervals for clinical and radiological assessment at 6, 12 weeks and at 6, 9, 12 months or longer if it was indicated.

The minimum follow-up was 12 months.

## Results

During the study period, 37 patients (14 male, 23 female) met the inclusion criteria and formed the basis of this review. Five patients (5/37, 13.5%) died within the first 6 months following discharge from hospital, and fracture union result could not be assessed. More specifically these were a 71M (43A3 open fracture grade IIIB) with metastatic breast cancer; an 81M (44B2 open fracture IIIA) with pneumonia, atrial fibrillation, cardiovascular disease and history of epilepsy; an 80F (44C3 open fracture IIIB) with metastatic sigmoid cancer, diabetes and hypertension; a 97F (44B3 closed fracture) with cardiac arrest, diabetes and depression; and 96F (44B4 open fracture II) with cardiac arrest and heart failure. Consequently, our cohort finally included 32 patients (12 males) with a mean age of 80.2 years (range 66–98) with a minimum follow-up of one 1 year (mean follow-up time 15.6 months (range 12–24).

In terms of the mechanism of injury, the vast majority of the patients sustained ground level falls (28/32, 87.5%) followed by road-traffic accidents (3/32, 9.4%) and fall from stairs (1/32, 3.1%). According to the AO classification, 22 were ankle {44A (1), 44B (16), 44C (5)} and 10 distal tibia fractures {43A (8), 43C (2)}. Eighteen open fractures were identified and classified according to the Gustilo–Anderson classification [11]. In particular, ten of them were type IIIB open fractures that needed additional plastic surgical interventions for soft tissue coverage, seven type II and one type IIIA. Skin flap was used in three cases, gracilis muscle flap in five cases and fasciocutaneous flap in two cases.

Moreover, 2 cases (2/32, 6.3%) were classified as polytrauma according to the New Berlin Definition [12] and their management was subject to ATLS and Damage Control Orthopaedics principles [13, 14], whereas 5 other cases (5/32, 15.6%) suffered from multiple injuries, not fulfilling the polytrauma criteria. The remaining 25 patients (25/32, 78.1%) sustained an isolated injury. Eleven fractures, eight open and three closed underwent staged procedure with initial stabilization with external fixation, given their bone comminution or soft tissue contamination.

**Fig. 1:**
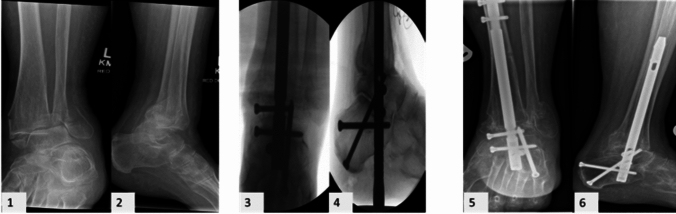
Closed isolated left comminuted ankle fracture in a female patient 87 years old sustained after a fall from standing height. (1, 2) preoperative AP and lateral radiographs of the ankle; (3, 4) intraoperative AP and lateral radiographs of the ankle demonstrating stabilization of the ankle and subtalar joint with the Phoenix Ankle Arthrodesis Nail; (5, 6) AP and lateral ankle radiographs showing ankle and subtalar fusion 4 months postoperatively

**Fig. 2:**
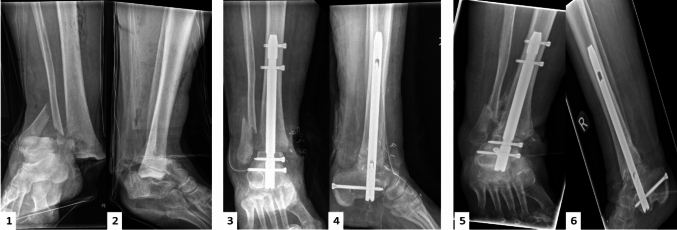
Isolated open ankle fracture dislocation in a female 85 years of age sustained after a fall from standing height with poor soft tissue conditions. (1, 2) preoperative AP and lateral radiographs of the ankle; (3, 4) immediate postoperative AP and lateral radiographs of the ankle demonstrating stabilization of the ankle and subtalar joint with the Phoenix Ankle Arthrodesis Nail; (5, 6) AP and lateral ankle radiographs showing ankle and subtalar fusion 8 months postoperatively

The mean time to definite management with the TTCN (Figs. 1, 2) was 7.4 days (range 0–36), whereas the mean postoperative hospitalization period was 28.8 days (range 2–180). Most patients were allowed to weight bear as tolerated immediately postoperatively (30/32, 93.8%), while instructions for restricted weight bearing were included in the rehabilitation protocol of 2 patients (2/32, 6.3%) due to the additional injuries that they suffered from or the comminuted pattern of their fracture.

Fracture union was uneventfully achieved in 30 patients (30/32, 93.8%) with a mean union time of 3.9 months (range 2–8), whereas in 2 patients (2/32, 6.3%) the union progress was restricted by the development of infection related to metalwork. One of these patients (diabetes, hypertension and depression/closed fracture 44B2) underwent nail removal and further management with fine-wire circular fixator 2 months post the TTCN procedure. The other one (heart failure, hypertension, atrial fibrillation and alcohol abuse/open IIIB 44C3 initially treated with a bridging external fixator) underwent amputation below the knee 3 months after the TTCN procedure.

Additionally, nail removal was performed in 3 cases (3/32, 9.4%) that developed surgical site infection after fracture union in 3, 4 and 8 months, respectively. One of these cases was a type II open fracture, while another developed hospital acquired pneumonia postoperatively. The last was a polytrauma patient that was initially managed with an external fixator and developed medial heel pin site infection. All cases were managed with removal of nail, canal debridement, irrigation and following tissue sample analysis with pathogen-specific systemic antibiotics for 6 weeks.

Considering the development of stress risers and the risk of fracture around the proximal aspect of the nail, one patient (1/32, 3.1%) proceeded to metalwork removal at 7 months postoperatively. Patient’s preference led to nail removal in only 1 case (1/32, 3.1%).

In 2 cases (2/32, 6.3%), osteolysis around the talus was noticed, but no reintervention was undertaken. The complication summary is shown in Table 1.

**Table 1:** Complications and reinterventions summary

Complication	N	Incidence (%)	Reinterventions
Infection before union	2	6.3	1 Nail removal + application of frame1 amputation
Infection after union	3	9.4	3 Nail removals
Stress risers development	1	3.1	1 Nail removal
Osteolysis around the talus	2	6.3	No reintervention
Total	8	25.1	6 Nail removals

Excluding the patient that developed sepsis and underwent amputation, no other systemic complication, such as thromboembolic events were recorded in our study.

Amongst the 30 patients that united, 9 patients completed the OMAS questionnaire. The rest were patients suffering from dementia or failed to comply with the OMAS patient-reported outcome scale questionnaire. The mean OMAS was 45 (range 20–70), suggesting that the treatment of the distal tibia and ankle fractures in our cohort, provided ‘fair’ functional outcomes, Table 2.

**Table 2:** Olerud–molander Ankle Score (OMAS)

	Age at the operation	Gender	AO classification	Additional injuries	OMAS
1	66	F	44B2	–	70
2	71	F	44C2	–	55
3	84	F	43C2	–	30
4	71	F	44C1	–	55
5	92	F	43A1	–	20
6	81	F	43A1	–	45
7	87	F	44B2	–	50
8	98	F	44B3	–	35
9	82	F	44B1	–	45
Mean	81.3				45

## Discussion

Lately, the benefit of minimal invasive surgery and immediate postoperative mobilization without restriction has been introduced for the treatment of osteoporotic ankle fractures with the use, of retrograde hindfoot nail [15]. The main indications for the use of such an implant have been the poor quality of their soft tissues, the overall frailty and poor preop state of the patients, usually above the age of 60 years, failure of previous conservative or operative treatment, complex fractures with severe bone loss or comminution and non-reconstructable pilon fractures [15, 16]. In our study, the same indications were mostly applied as well as the preinjury restricted ambulation, i.e. wheelchair bound or house-bounded and with mental disorders being unable to follow instructions of mobilization during the postoperative period. It should be noted that the primary endpoint of this study was to evaluate the fracture union, as joint fusion was not the primary goal in such cases.

The shorter hospitalization period and reduced risk of postoperative complications [17] are not depicted in our study, since the mean postoperative length of stay was 28.8 days, ranging from 2 to 180 days. Prolonged stay was attributed for five patients to the development of hospital acquired pneumonia and for three patients to persistent urinary tract infections. Despite the allowance of immediate weight bearing following TCCN in the majority of our patients exempting only those with additional injuries, the average hospital stay was prolonged. The main reasons for that were related to care home bed availability and other care package delays.

Noteworthy, due to the advanced age and the multiple comorbidities of the patients who are managed with the retrograde hindfoot nail, the mortality rate has been reported to be impressively high, fluctuating from 25 to 46% [18, 19]. In the herein study, of the five deaths during the follow-up period, two had metastatic cancer, two were older than 95 years and one had heavy past medical history, forming a mortality rate of 13.5%, significantly lower than other studies [17, 18].

Regarding the clinical outcomes, union was achieved in 30 out of 32 patients. Further to our experience, the results from the use of TTCN for the stabilization of distal tibia and ankle fractures have been encouraging with high union rates (up to 100%) in some studies [19–21], while a systematic review points out a non-union rate of 13.3% [22]. The latter concluded that given the quality of life and the demands that the patients who undergo talotibiocalcaneal fixation have, non-unions do not require implant removal, when asymptomatic.

However, in another study, 11% of the cases required metalwork removal or further procedures to enhance union [23]. In our cohort, 2 out of 32 cases developed early infection and required further intervention before union occurrence. In one patient, the infection resulted to non-union (nail removal in the second postoperative month and stabilization with Ilizarov construct), whereas the other became septic and underwent amputation 3 months postoperatively.

Interestingly, during the follow-up period, stress bone formation was noticed radiographically around the proximal aspect of the Phoenix nail in one of the patients. This condition was accompanied by osteolysis around the locking screws of the nail, so the nail was removed in 7 months to avoid the risk of the development of periprosthetic fracture. Thomas et al. demonstrate that the accumulation of the bending forces at the proximal end of the nail can result in stress fracture, which can be suspected with local pain 6–8 weeks postoperatively [24]. Jonas et al. support that the periprosthetic fracture is the result of the modulus mismatch between the tip of the nail and the canal of the tibia [21]. A biomechanical analysis of the effect of nail length concluded that the problem of periprosthetic fractures can be overpassed with the use of implants that rest in the proximal tibial metaphysis, 5 cm below the knee articulation [25]. Some authors have adopted this principle and do not report any periprosthetic fractures in their studies [4, 20]. Routine implant removal after union has been a controversial topic with some authors supporting it to avoid the devastating complication of the fracture [19], while others vote against it, considering the cost of a second intervention in a medically unstable host [21]. We recommend the removal of the nail in any fit for surgery patient with pretibial pain or radiographic features of stress reaction.

The incidence of infection in this series was 15.7%. Two cases of infection required nail removal before union, whereas in the other 3 as the infection occurred after union, the nail was removed without any further interventions. This rate is comparable to the results of Taylor et al. [26], but slightly lower when compared to the results of a systematic review conducted by Lu [27], who reported that infection appeared to 18% of the cases. We also found a high incidence of reoperation rate (21.9%) as in 7 cases out 32, the nail was removed, either required (18.8%) or not (one prearranged removal). In a recent systematic review by Tan [28], the unplanned return to the operating room occurred in 10.1% of the 252 ankle fractures.

In terms Olerud–Molander Ankle Score as this is the most commonly used score in the literature and would have allowed us to compare our results to other published studies. Lemon et al. published the highest mean score (69.6) in their study with 12 patients, followed by Georgiannos et al. (score 61 in 43 patients), while our score was 45 (Table 3). Another recent similar case series in a major trauma centre with 20 patients [29] demonstrated mean OMAS 50.9. The differences noted in the scores could be related to different demographically patient controls and the small number of patients studied.

**Table 3:** Comparison of functional outcomes

Author	Sample size	Age	Fracture type	OMAS score
Lemon et al. [19]	12	84	Ankle	69.6
Amirfeyz et al. [18]	13	79	Ankle	50
Jonas et al. [21]	31	77	Ankle	45
Al-Nammari et al. [20]	48	82	Ankle	57
Georgiannos et al. [17]	43	78	Ankle	61
Lu et al. [29]	20	77	Ankle	50.9
Our study	9	81.3	Ankle and distal tibia	45

The retrospective design of this study, as well as the small sample of patients, constitutes the main limitations of the study. Strengths include the consecutive patient recruitment, the length of the recruitment period from a large university major trauma centre clinic indicating that TTCN nail is performed infrequently being a salvage procedure and thus the small number of patients recruited. The findings of this study can constitute the base of the design for future studies and reviews.

## Conclusion

The treatment of complex distal tibia and ankle fractures has been challenging, especially when it comes to elderly patients with poor bone quality or compromised skin condition. Potential non-compliance to postoperative instructions or mental health disorders can maximize the challenges and highlights the need of a treatment solution that can address all these challenges. In our study, the hindfoot nail has showed good results towards this direction, facilitating early mobilization with full weight bearing and good union rate. More studies are desirable to establish the superiority of tibiotalocalcaneal nails for the management of such cases.
